# Exploiting the aerobic endospore-forming bacterial diversity in saline and hypersaline environments for biosurfactant production

**DOI:** 10.1186/s12866-015-0575-5

**Published:** 2015-10-28

**Authors:** Camila Rattes de Almeida Couto, Vanessa Marques Alvarez, Joana Montezano Marques, Diogo de Azevedo Jurelevicius, Lucy Seldin

**Affiliations:** Laboratório de Genética Microbiana, Instituto de Microbiologia Paulo de Góes, Universidade Federal do Rio de Janeiro, Centro de Ciências da Saúde, Bloco I, Ilha do Fundão, Rio de Janeiro, RJ CEP 21941-590 Brazil

**Keywords:** Biosurfactants, Endospore-forming bacteria, Saline and hypersaline environments, Microbial enhanced oil recovery, Bioremediation

## Abstract

**Background:**

Biosurfactants are surface-active biomolecules with great applicability in the food, pharmaceutical and oil industries. Endospore-forming bacteria, which survive for long periods in harsh environments, are described as biosurfactant producers. Although the ubiquity of endospore-forming bacteria in saline and hypersaline environments is well known, studies on the diversity of the endospore-forming and biosurfactant-producing bacterial genera/species in these habitats are underrepresented.

**Methods:**

In this study, the structure of endospore-forming bacterial communities in sediment/mud samples from Vermelha Lagoon, Massambaba, Dois Rios and Abraão Beaches (saline environments), as well as the Praia Seca salterns (hypersaline environments) was determined via denaturing gradient gel electrophoresis. Bacterial strains were isolated from these environmental samples and further identified using 16S rRNA gene sequencing. Strains presenting emulsification values higher than 30 % were grouped via BOX-PCR, and the culture supernatants of representative strains were subjected to high temperatures and to the presence of up to 20 % NaCl to test their emulsifying activities in these extreme conditions. Mass spectrometry analysis was used to demonstrate the presence of surfactin.

**Results:**

A diverse endospore-forming bacterial community was observed in all environments. The 110 bacterial strains isolated from these environmental samples were molecularly identified as belonging to the genera *Bacillus, Thalassobacillus, Halobacillus, Paenibacillus, Fictibacillus* and *Paenisporosarcina*. Fifty-two strains showed emulsification values of at least 30%, and they were grouped into18 BOX groups. The stability of the emulsification values varied when the culture supernatants of representative strains were subjected to high temperatures and to the presence of up to 20% NaCl. The presence of surfactin was demonstrated in one of the most promising strains.

**Conclusion:**

The environments studied can harbor endospore-forming bacteria capable of producing biosurfactants with biotechnological applications. Various endospore-forming bacterial genera/species are presented for the first time as biosurfactant producers.

## Background

Aerobic endospore-forming bacteria are ubiquitous members of microbial communities in various environments. Spores ensure the survival of bacteria through long periods of harsh conditions [1]. Endospore-forming bacteria are of particular interest because of their biotechnological potential such as the production of industrially important enzymes and bioactive chemicals, the biological degradation of pollutants and their use as biopesticides. Their roles in remediation, plant-growth promotion, biological control and other applications have been thoroughly presented by Mandic-Mulec and Prosser [2].

Biosurfactants are one of the bioactive chemicals produced by aerobic endospore-forming bacteria. They are usually produced to the extracellular medium, and some of them have the unique property of reducing the surface and interfacial tension of liquids. They are also important due to their environmentally friendly nature, selectivity and potential for large-scale production. Biosurfactants have applications in the fields of agriculture, biomedical sciences, cosmetics, food processing, pharmaceuticals, and in the petrochemical industry. For example, their use in microbial-enhanced oil recovery (MEOR) is one of the most promising fields for biosurfactant application [3–5]. However, the biosurfactants must maintain their activity under the physical and chemical conditions (such as high temperatures and salinities) that they would be exposed to during the MEOR process in oil reservoirs.

Among the biosurfactants produced by the aerobic endospore-forming bacteria, surfactin and lichenysin are two well-studied lipopeptide surfactants produced by *B. subtilis* and *B. licheniformis*, respectively [4]. They show effectiveness under extreme temperature and pH conditions [6, 7] and exhibit a broad spectrum of activities that confer them a great potential for development in bioprocesses [8].

Potent biosurfactant-producing *Bacillus* species from natural habitats have been reported [5], and various studies have focused on the isolation of more efficient surfactin (and other biosurfactants) producers. As an example, some researchers have been investing in approaches such as the search for surfactin producers in extreme habitats [9] and the development of novel methods for the rapid screening of these producers in various environments [2, 10]. However, very little is known about the biosurfactants produced by other members of the group of endospore-forming bacteria.

The ubiquity of endospore-forming bacteria in saline and hypersaline environments is well known. These halotolerant or halophilic endospore-forming bacteria may offer a multitude of actual or potential applications in various fields of biotechnology such as oil recovery [11, 12]. However, studies on the diversity of biosurfactant-producing and endospore-forming bacterial genera/species in saline and hypersaline habitats are underrepresented. The search for biosurfactant producers within halophilic/halotolerant bacteria appears to be particularly promising because the biosurfactants of these organisms may have adaptations that can increase their stability in adverse environments. Therefore, in this study, saline and hypersaline environments were chosen for the isolation of potential biosurfactant producers belonging to the endospore-forming bacterial group in an attempt to discover biosurfactants that are stable in high concentrations of salt. Their stability at high temperatures was also considered. Firstly, the structure of aerobic endospore-forming bacteria in these environmental samples was molecularly compared and correlated with the differences in salinity to select environments not only containing various concentrations of salt but also various endospore-forming bacterial communities.

## Methods

### Sample sites

Sediment samples (50 g, 0 to 10 cm deep) from Dois Rios Beach (DR), Abraão Beach (AB), Massambaba Beach (MA) (marine ecosystems), Vermelha Lagoon (VM), two different salterns (SA1 and SA2), as well as a mud sample (LS) from one of the salterns (hypersaline ecosystems) were collected in triplicate. The permission for sampling was given by “Instituto Estadual do Ambiente” – INEA 015/2015. DR and AB are located in Ilha Grande State Park, Angra dos Reis, Rio de Janeiro, and MA, VM, SA1, SA2 and LS are located in the Massambaba Environmental Protection Area, Saquarema, Rio de Janeiro, Brazil. The location and the physical, chemical and environmental proprieties of the samples are described in Table 1. Other features of the Vermelha Lagoon and Massambaba Beach were previously described in Jurelevicius et al. [13].

**Table 1:** Physicochemical properties of the sediment and mud samples used in the study

Samples	Coordinates	Water temperature	pH	Salinity (%)
Dois Rios Beach	23°11‘S 44°11‘W	24 °C	7.00	3.5
Abraão Beach	23°08‘S 44°09‘W	24 °C	7.50	3.6
Massambaba Beach	22°57‘S 42°19‘W	24 °C	8.00	4.0
Vermelha Lagoon	22°55‘S 42°23‘W	28 °C	8.05	5.0
Saltern 1	22°54‘S 42°19‘W	35 °C	8.16	12
Saltern 2	22°54‘S 42°19‘W	39 °C	7.37	27
Saltern 2 (mud)	22°54‘S 42°19‘W	39 °C	7.00	27

### DNA extraction

Total microbial community DNA was extracted directly from the sediment or mud samples (0.5 g of each sample in triplicate) using the FastDNA® Spin Kit for Soil (MP Biomedicals, Santa Ana, CA, USA). DNA preparations were visualized via electrophoresis in 0.8 % agarose gel in 1x TBE buffer [14] and then stored at 4 °C prior to PCR amplification. The amount of DNA extracted from each sample was determined using a NanoDrop 1000 spectrophotometer (Thermo Scientific, Suwanee, GA, USA).

### PCR amplification of spore-forming bacterial 16S rRNA encoding gene

A semi-nested PCR was used to amplify the 16S rRNA encoding gene (*rrs*) of spore-forming bacteria. The first reaction was performed using the pair of primers BacF [15] and L1401 [16] in a 25-μl mixture containing approximately 10 ng of DNA, 100 nM of each primer, 0.2 mM of each dNTP, 2.5 mM MgCl_2_, 1.25 U *Taq* DNA polymerase (Promega, Madison, WI, USA) and 5 μl of 5X PCR buffer supplied by the manufacturer. The amplification conditions were as follows: initial denaturation for 5 min at 94 °C; 35 cycles of 1 min at 94 °C, 1 min at 58 °C, and 2 min at 72 °C; a final extension for 10 min at 72 °C; and cooling to 4 °C. Amplicons obtained in this first PCR reaction were then used as templates for a second amplification with primers 968 F-GC and R1401 [16]. The reaction mixture was the same as described above with the exception of MgCl_2_ (3.75 mM used). The amplification conditions were initial denaturation for 5 min at 94 °C; 30 cycles of 1 min at 94 °C, 1 min at 55 °C, and 2 min at 72 °C; a final extension for 10 min at 72 °C; and cooling to 4 °C.

### Denaturing gradient gel electrophoresis (DGGE) and statistical analyses

DGGE analysis was carried out as previously described [17] using the Ingeny PhorU2 apparatus (Ingeny International BV, The Netherlands). PCR products were loaded onto 8 % (w/v) polyacrylamide gels in 1X TAE buffer (20 mM Tris-acetate, pH 7.4, 10 mM acetate, 0.5 mM disodium EDTA). The polyacrylamide gels contained a denaturing gradient of urea and formamide varying from 46.5 to 60 %. The gels were run for 17 h at 140 V and 65 °C. After this period, they were soaked stained for 1 h in SYBR Green I nucleic acid staining solution (1.000X concentrated; Molecular Probes, The Netherlands) and immediately photographed under UV light. Dendrograms were constructed based on the presence and absence of bands with the unweighted pair group method (UPGMA) with mathematical averages and similarity coefficient of Dice using the BioNumerics software (Applied Maths, Ghent, Belgium). Additionally, the binary matrices generated from the DGGE lanes were exported to PAST software [18] for Non-metrical Multidimensional Scaling (NMDS) analysis.

### Isolation of endospore-forming bacteria

For the isolation of the endospore-forming bacteria, 1 g of each sediment/mud sample was resuspended in saline (9 ml), shaken for 20 min at room temperature and then heated to 80 °C for 10 min. Serial decimal dilutions were subsequently plated in triplicate onto trypticase soy broth (TSB), TSB + NaCl 3.5 %, TSB + NaCl 7 % and Marine broth (MB, Difco - containing high salt content and numerous minerals that duplicate the major mineral composition of sea water), all supplemented with 1.2 % agar. Plates were incubated at 32 °C for up to 7 days. The bacterial population density was estimated through the determination of colony-forming units (CFU) in TSB agar plates (for non-halophiles and/or halotolerant bacteria) and in MB agar plates (for halotolerant and/or halophilic bacteria). Statistical analysis for CFU counts was performed using Tukey’s test (*p* < 0.05). Colonies presenting different morphological characteristics in each plate used (containing various amounts of salt) were selected for further purification. Bacterial pure cultures were stored at −80 °C in the same medium in which they were isolated with 10 % glycerol.

The isolates were designated using the initials of the place in which they were isolated (VM – Vermelha Lagoon, DR – Dois Rios Beach, MA – Massambaba Beach, AB – Abraão Beach, SA1 – Saltern 1, SA2 – Saltern 2 and LS – Saltern 2 mud), followed by progressive numbers of isolation.

### 16S rRNA sequence analysis and identification

The isolates were grown in the same culture medium from which they were isolated at 32 °C for 48 h, and the ZR Fungal and Bacterial DNA MiniPrep (Zymo Research, Irvine, CA, USA) was used according to the manufacturer’s instructions for their DNA extraction. 16S rRNA gene amplification (1,562 bp) was performed using the primers PA (5′ AGAGTTTGATCCTGGCTCAG 3′) and PH (5′AAGGAGGTGATCCAGCCGCA 3′), as described by Massol-Deya et al. [19]. Further, the PCR-amplified 16S rRNA gene of each isolated bacteria were re-amplified and sequenced using the universal primers 518 F (5′CCAGCAGCCGCGGTAATACG3′) and the facilities of Macrogen (Korea). The 16S rRNA sequences obtained were compared with the sequences previously deposited at the GenBank database using the BLAST-N facility (www.ncbi.nlm.nih.gov/blast). For phylogenetic tree analyses, the sequences of closely related bacterial strains were recovered from GenBank database and aligned to the sequence obtained in this study using the CLUSTAL-X software [20]. BioEdit version 7.0.5.3 (www.mbio.ncsu.edu/Bioedit/bioedit.html) was used for manual editing of the sequences. Phylogenetic trees were constructed based on 16S rRNA sequences via the unweighted pair group method with the arithmetic mean (UPGMA) method [21] using MEGA 5 software [22]. The sequences obtained in this study are available in GenBank under the accession numbers KR133283 - KR133392.

### Screening for biosurfactant production

#### Emulsification assay

The emulsification assay used was previously described by Cooper and Goldenberg [23]. Cell-free culture broth (200 μl) obtained as described above was used to determine the emulsification of either kerosene or hexadecane. In an Eppendorf tube containing 600 μl of distilled water, 1.2 ml of kerosene/hexadecane was mixed with each bacterial supernatant in triplicate. The mixture was vortex-shaken for 2 min, and the emulsion was allowed to stand for 24 h. A negative control was performed only with water and kerosene or hexadecane. After the period of 24 h, the height of the emulsion layer was measured. The emulsification index was calculated based on the ratio of the height of emulsion layer and the total height of the liquid [EI % = (emulsion/ total h) x 100]. To determine the stability of the emulsification ability of the biosurfactant, the emulsification index was also determined after 15 and 30 days. The strains that showed emulsification indexes higher than 30 % were selected for further studies.

#### Drop-collapse test

A drop-collapse test was performed using all isolates following the procedure described by Jain et al. [24]. Strains were grown in TSB and/or MB medium for 7 days, and 5 μl of the cell-free culture broth (supernatant centrifuged 13.000 x g, 15 min) was dropped on a glass slide covered with crude oil. The result was considered positive for biosurfactant production when the drop diameter was at least 1 mm larger than that produced by distilled water (negative control).

#### Oil spreading assay

For this assay, 10 μl of crude oil was added on the surface of a Petri dish containing 40 ml of distilled water to form a thin layer of oil, as described in Morikawa et al. [25]. Culture supernatants (10 μl, obtained as above) were then placed in the center of the oil layer. If biosurfactant is present in the supernatant, the oil will be displaced with an oil-free clearing zone. A negative control was performed with distilled water (without biosurfactant), and no oil displacement or clear zone was observed.

### Hemolytic assay

A hemolytic assay was performed using all isolates in 5 % sheep blood agar plates as described by Mulligan et al. [26]. The cultures were spot-inoculated onto plates containing blood agar and incubated at 32 °C for 48 h. The plates were visually inspected for the formation of a clear zone (hemolysis) around the bacterial spots.

### Production of antimicrobial substance (AMS)

The method described by Rosado and Seldin [27] was used to detect the production of AMS. All isolated strains were grown in TSB at 32 °C for 24 h and then spotted (5 μl) on the surface of TSB and/or MB agar plates that were incubated for 7 days at 32 °C. After incubation, the cells were killed by exposure to chloroform vapor for 30 min. *Micrococcus* sp. was used as the indicator strain as described in von der Weid et al. [28, 29]. The production of AMS was suggested by the presence of a clear zone of growth inhibition around the bacterial spots.

### PCR screening for *sfp* gene

Screening for the presence of the *sfp* gene from *B. subtilis* encoding 4′-phosphopantetheinyl transferase was carried out using the primers sfpF- 5′ ATGAAGATTTACGGAATTTA 3′ and sfpR- 5′ TTATAAAAGCTCTTCGTACG 3′, which amplify a 675-bp fragment of the surfactin gene [30]. The PCR reaction was performed in the following mix: 1 μl (50–100 ng) of target DNA, 5 μl of 5X PCR buffer (Promega, RJ, Brazil), 3.75 mM MgCl_2_, 0.5 mM dNTP, 1 μM of each PCR primers and 1.25 U of *Taq* polymerase in a 25-μl final volume. Thermal cycling was carried out with an initial denaturation of 94 °C for 5 min followed by 35 cycles at 94 °C for 40 s, 48 °C for 1 min, and 72 °C for 40 s, followed by a final extension of 72 °C for 10 min. The PCR-amplified *sfp* gene of the different isolates was re-amplified and sequenced using the primers sfpF and Macrogen (Korea) facilities. The sequences obtained were compared with the sequences previously deposited in the GenBank database using the BLAST-N facility (www.ncbi.nlm.nih.gov/blast).

### BOX-PCR

Amplification reactions using the primer BOXA1R [31] were performed in the following mix: 1 μl (50–100 ng) of target DNA (from the strains that showed emulsification values higher than 30 %), 5 μl of 5X PCR buffer (Promega, RJ, Brazil), 3.75 mM MgCl_2_, 0.5 mM dNTP, 1 μM of the primer BOXA1R, and 1.25 U of *Taq* polymerase in a 25-μl final volume. The amplification conditions were as follows: initial denaturation for 7 min at 94 °C; 35 cycles of 1 min at 94 °C, 1 min at 53 °C, and 8 min at 72 °C; a final extension for 10 min at 72 °C; and cooling to 4 °C. Negative controls (without DNA) were run during all amplifications. Agarose gel electrophoresis of the PCR products was performed using 1.4 % agarose in 1X TBE buffer at 90 V for 4 h at room temperature. The BOX results were collected into matrices indicating the presence or absence (scored as 1 or 0, respectively) of bands. Dendrograms were constructed using Dice similarity coefficients and the unweighted pair group method with arithmetic mean (UPGMA) with the BioNumerics software package (Applied Maths, Ghent, Belgium).

### Biosurfactant stability after salt and heat treatments

Strains presenting emulsion values higher than 30 % were used for both tests. For salt stability, the emulsification assay was repeated by replacing the water with different concentrations of saline solutions (NaCl 5 %, 10 %, 15 % and 20 %). For heat resistance, the culture supernatants were heated to 100 °C and 121 °C and then submitted to the emulsification assay. All tests were performed in triplicate using hexadecane and kerosene, and the emulsion indexes were calculated after 1, 15 and 30 days.

### Crude biosurfactant extraction and mass spectrometry analysis

The supernatant of different biosurfactant-producing strains was separated from bacterial cells as described above and acidified with HCl to pH 2. The extraction was performed with chloroform and methanol (2:1 – v/v) as described by Korenblum et al. [32]. The crude extracts were analyzed via direct infusion using an ESI–MS/MS triple quadrupole mass spectrometry (API 2000 LC/MS/MS System - AB SCIEX, Massachusetts, USA) operating in the negative ion mode. Commercial surfactin (Sigma Aldrich) was used as a positive control.

## Results

### Distribution of the endospore-forming bacteria

**Fig. 1:**
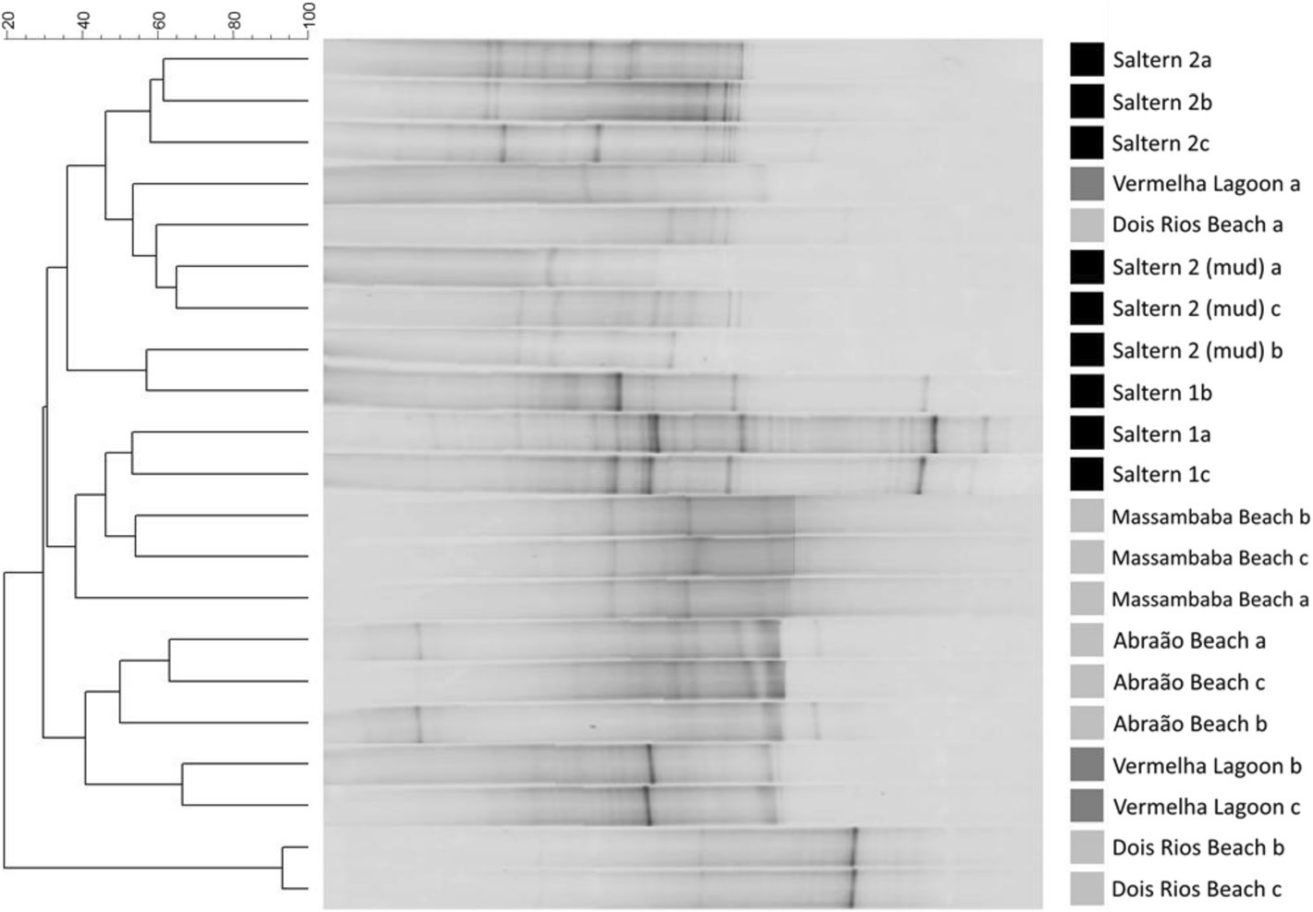
Denaturing gradient gel electrophoresis fingerprint and UPGMA cluster analysis based on16S rRNA coding gene fragments of the endospore-forming bacterial communities in the various marine and hypersaline environments sampled in Ilha Grande State Park, Angra dos Reis and the Massambaba Environmental Protection Area, Saquarema, Rio de Janeiro

PCR-DGGE was used to analyze the structure and distribution of endospore-forming bacterial communities in various marine and hypersaline environments (Fig. 1). The results showed the presence of a complex endospore-forming bacterial community in all environments. In general, the triplicate samples of each environment showed great reproducibility of band patterns; however, some outliers were observed. A dendrogram generated after DGGE analyses confirmed the reproducibility of the triplicates and grouped the samples in clusters. For example, the endospore-forming bacterial communities from hypersaline samples containing more than 20 % salinity (Saltern 2 and Saltern 2 mud) were clustered with more than 40 % similarity. However, endospore-forming bacterial communities present in two samples of Dois Rios Beach shared less than 20 % similarity with endospore-forming bacterial communities present in all other environments studied.

**Fig. 2:**
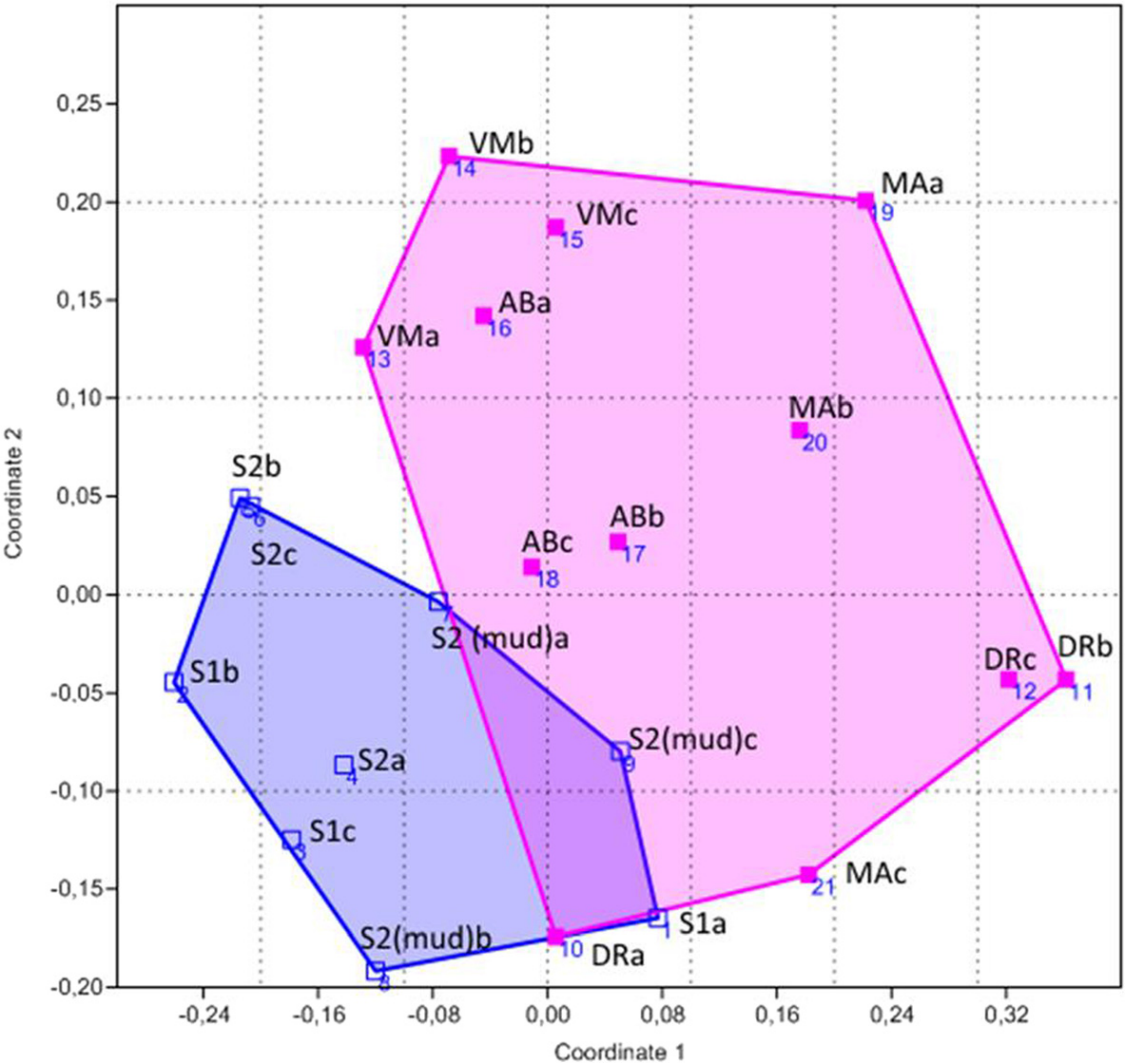
NMDS ordination diagram based on the genetic fingerprint data of the endospore-forming bacterial communities. The diagram corresponds to the genetic fingerprint pattern presented in Fig. 1

NMDS analyses confirmed the tendency of clustering the samples in two groups based on the salinity of the environments: (i) the samples from environments with salinity ranging from 3.5 to 5.0 % and (ii) samples from environments with salinity ranging from 12 to 27 % (Fig. 2). The structural diversity of endospore-forming bacterial communities in the different marine and hypersaline environments studied here makes these environmental samples hotspots for the isolation of potential biosurfactant producers belonging to this bacterial group.

### Enumeration and isolation of endospore-forming bacteria

**Fig. 3:**
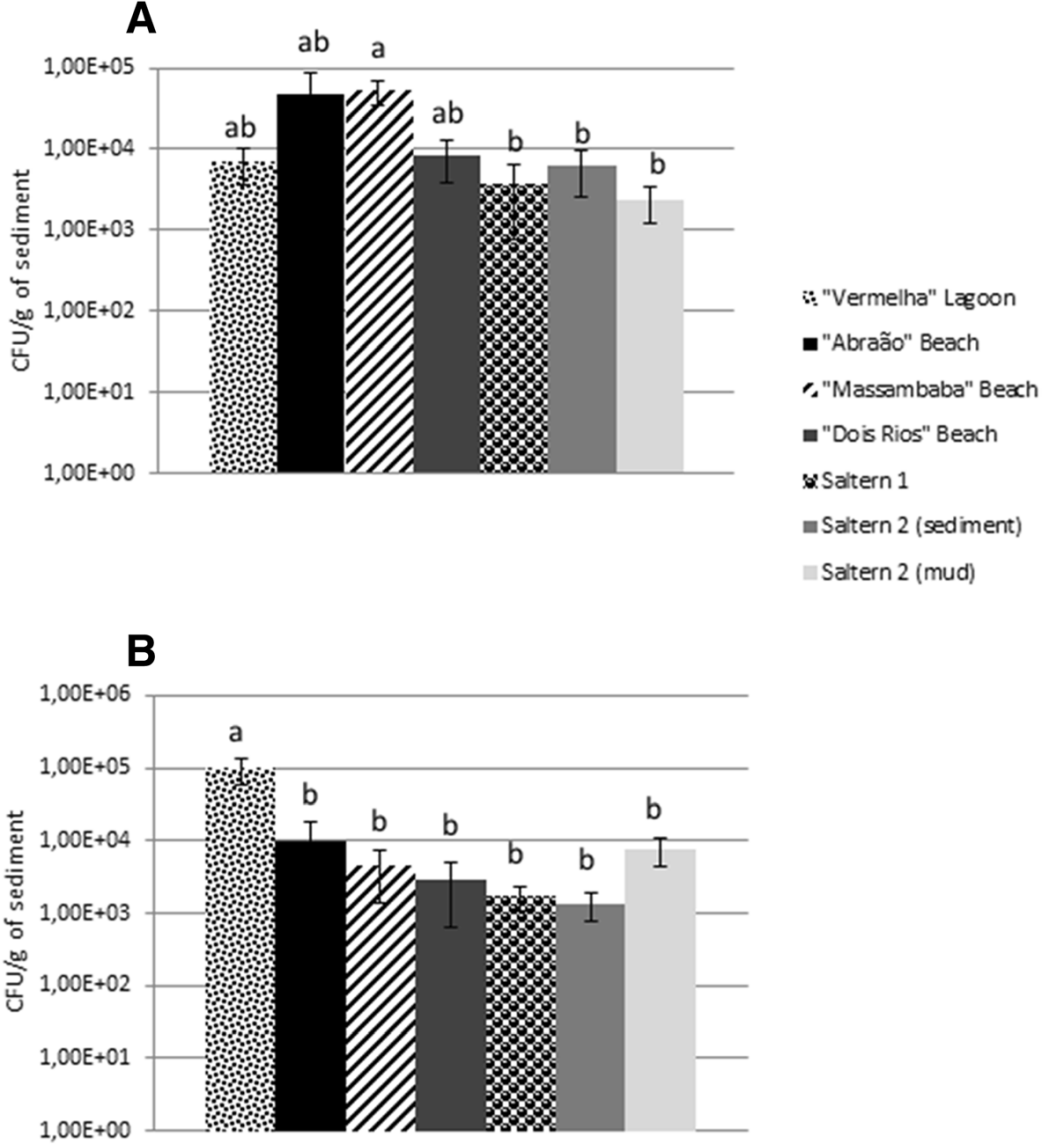
Enumeration of halotolerant and halotolerant/halophilic endospore-forming bacteria using TSB (**a**) and MB agar (**b**) media. Means with different letters are significantly different (Tukey test, *p* < 0.05)

Non-halophiles/halotolerant and halotolerant/halophilic endospore-forming bacterial enumerations were performed using TSB and MB agar media, respectively (Fig. 3). The number of endospore-forming bacteria varied from 1.3x10^3^ CFU g^−1^ (Saltern 2) to 9.5x10^4^ CFU g^−1^ (Vermelha Lagoon), both in MB agar medium. The CFU was usually lower in MB agar medium compared with TSB agar, with the exception of that from Vermelha Lagoon. Significant differences (Tukey’s test, *p* <0.05) were observed between CFU quantification in Massambaba and Saltern 1, Saltern 2 and Saltern 2 mud (TSB) and between the Vermelha Lagoon and the remaining samples (MB) (Fig. 3).

**Fig. 4:**
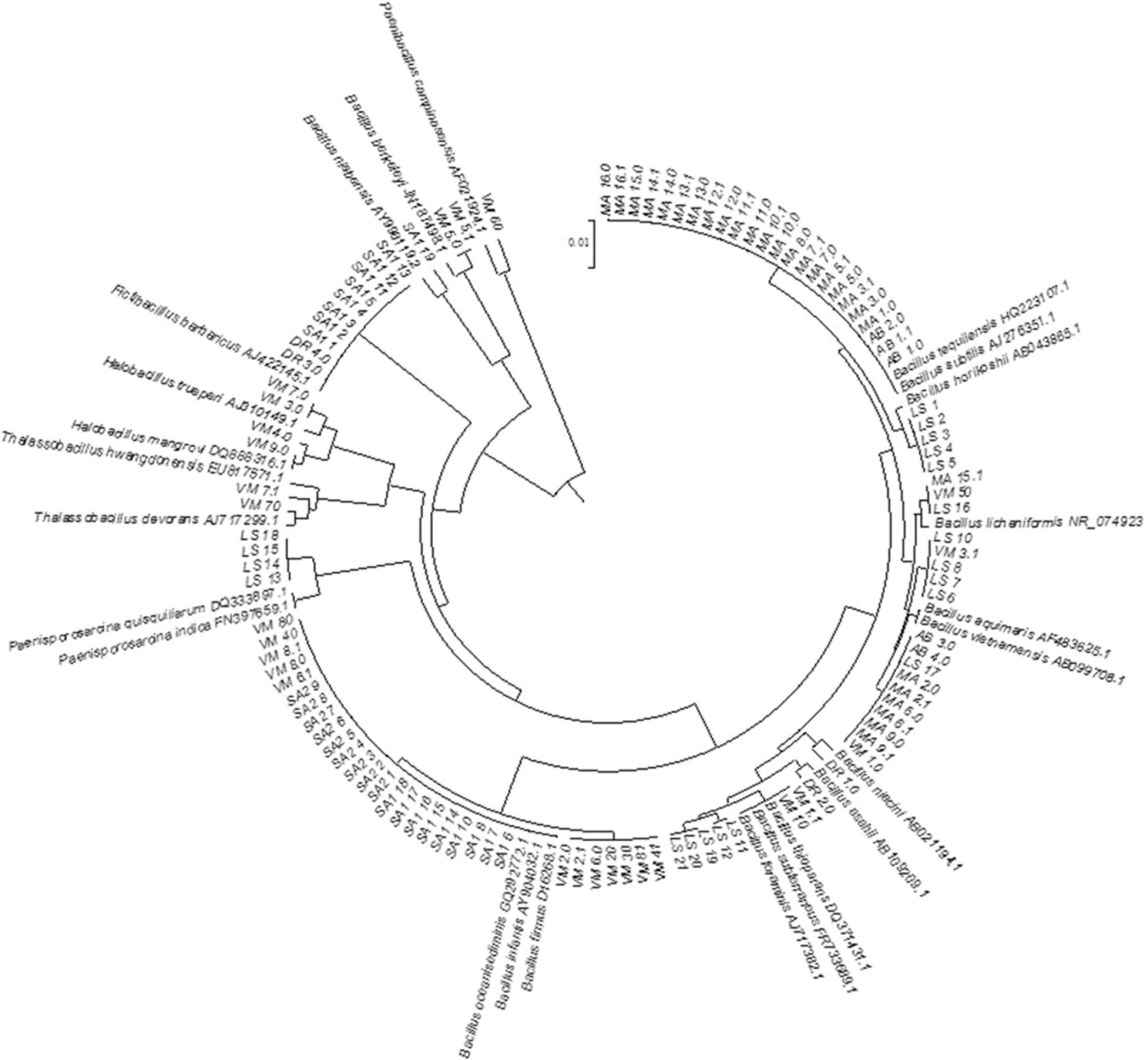
Phylogenetic tree of 16S rRNA gene sequences (approximately 800 bp) showing the relationship among the endospore-forming bacterial isolates. The tree was constructed based on the unweighted pair group method with arithmetic mean (UPGMA). Numbers on nodes represent bootstrap values with 1,000 repetitions

The isolation of halotolerant and halotolerant/halophilic endospore-forming bacteria was performed using TSB agar, TSB agar with the addition of 3.5 or 7 % NaCl and MB media. After 7 days of culture incubation at 32 °C, 110 endospore-forming bacterial strains were obtained. Among them, 26 endospore-forming strains were isolated from Vermelha Lagoon, 4 strains from Dois Rios Beach, 28 strains from Massambaba Beach, 5 strains from Abraão Beach, 18 strains from Saltern 1, 9 strains from Saltern 2 and 20 strains from Saltern 2 mud (Table 2). All of these strains were further identified by partial sequencing of the gene coding for 16S rRNA. Different endospore-forming bacterial genera were detected in saline and hypersaline samples, and 80.9 % of these bacterial strains belonged to genera *Bacillus* (isolated from all samples), 9.1 % to *Fictibacillus* (from Dois Rios Beach and Saltern 1), 3.6 % to *Halobacillus* (from Vermelha Lagoon), 0.9 % *Paenibacillus* (from Vermelha Lagoon), 3.6 % *Paenisporosarcina* (Saltern 2 mud) and 1.8 % *Thalassobacillus* (from Vermelha Lagoon) (Table 2) when the first hit using BLASTn was considered. As observed here, Vermelha Lagoon has the most diverse endospore-forming community among the environments studied. Based on the phylogenetic tree constructed (Fig. 4), *B. oceanisediminis* - *B. firmus* as well as *B. subtilis* - *B. tequilensis* could not be separated. These species predominated among the strains belonging to the genus *Bacillus* (Table 2, Fig. 4). *Bacillus horikoshii*, *B. vietnamensis*, *Paenisporosarcina quisquiliarum* and *P. indica* were isolated only from the Saltern 2 mud in which the amount of salt reached 27 % (Tables 1 and 2).

**Table 2:** Strains isolated from different environments, their molecular identification and biosurfactant production and other characteristics

Strains	Isolation media	Identification (% identity) – accession number ^a^	BOX group	E24-Hex			E24-Ker			DC	OD (cm)	H	*sfp*	AMS
				(EI %)			(EI %)							
				1 day	15 days	30 days	1 day	15 days	30 days					
Vermelha Lagoon														
VM 1.0	MB	*Bacillus aquimaris* (99) - JF411268.1		27 ± 2.3	-	-	-	-	-	-	-	-	-	-
VM 1.1	MB	*B. subterraneus* (100) - KJ722431.1		-	-	-	-	-	-	-	-	-	-	-
VM 2.0	MB	*B. infantis* (99) - KJ911913.1		-	-	-	-	-	-	-	-	-	+	+
VM 2.1	MB	*B. infantis* (100) - KJ911913.1		10 ± 0	8 ± 0	-	24 ± 5.7	16 ± 8	12 ± 5.7	+	-	-	+	+
VM 3.0	MB	*Halobacillus trueperi* (99) - KJ009504.1		-	-	-	-	-	-	-	-	-	-	-
** VM 3.1**	MB	*B. vietnamensis* (100) - JF411279.1	14	38 ± 6.9	38 ± 6.9	38 ± 6.9	-	-	-	+	-	-	-	+
VM 4.0	MB	*H. mangrovi* (99) - JQ799099.1		-	-	-	-	-	-	-	-	-	-	-
VM 5.0	MB	*B. berkeleyi* (99) - NR109459.1		-	-	-	-	-	-	-	-	-	-	-
VM 5.1	MB	*B. berkeleyi* (99) - NR109459.1		-	-	-	-	-	-	-	-	-	-	-
** VM 6.0**	MB	*B. infantis* (99) - KJ911913.1	15	19 ± 2.3	15 ± 1.2	15 ± 1.2	34 ± 2.8	27 ± 9.2	13 ± 8.3	-	-	-	+	+
** VM 6.1**	MB	*B. infantis* (99) - KJ911913.1	16	47 ± 5.8	24 ± 6.9	21 ± 6.1	8 ± 83.5	6 ± 0	-	-	-	-	-	-
VM 7.0	MB	*H. trueperi* (99) - KJ009504.1		-	-	-	-	-	-	-	-	-	+	-
VM 7.1	MB	*Thalassobacillus hwangdonensis* (100) - NR104552.1		-	-	-	-	-	-	-	-	-	-	-
** VM 8.0**	MB	*B. infantis* (99) - KJ911913.1	9	19 ± 1.2	19 ± 1.2	15 ± 1.2	30 ± 2.8	17 ± 1.2	11 ± 1.2	-	-	-	-	-
VM 8.1	MB	*B. infantis* (99) - KJ911913.1		21 ± 1.2	21 ± 1.2	21 ± 1.2	-	-	-	+	-	+	-	-
VM 9.0	MB	*H. mangrove* (99) - JQ799099.1		-	-	-	-	-	-	-	-	-	+	-
VM 10	TSB + 3.5 % NaCl	*B. subterraneus* (99) - KJ722431.1		-	-	-	-	-	-	-	-	-	+	-
** VM 20**	TSB + 3.5 % NaCl	*B. infantis* (99) - KJ911913.1	17	18 ± 3.5	12 ± 4	12 ± 4	32 ± 5.7	-	-	-	-	-	-	+
** VM 30**	TSB	*B. infantis* (99) - KJ911913.1	4	33 ± 2.3	33 ± 2.3	30 ± 0	36 ± 2.8	28 ± 5.7	20 ± 5.7	+	-	-	-	-
** VM 40**	TSB	*B. infantis* (99) - KJ911913.1	10	23 ± 2.3	23 ± 2.3	23 ± 2.3	36 ± 2.8	31 ± 9.5	31 ± 9.5	-	-	-	-	-
** VM 41**	TSB	*B. infantis* (99) - KM077280.1	4	17 ± 6.1	16 ± 7.2	16 ± 7.2	32 ± 2.8	24 ± 3.5	22 ± 3.5	-	-	-	-	-
** VM 50**	TSB	*B. licheniformis* (100) - LN774355.1	12	-	-	-	31 ± 2.3	-	-	-	-	+	-	+
VM 60	TSB	*Paenibacillus campinasensis* (99) - JF830004.1		-	-	-	-	-	-	-	-	-	-	-
VM 70	TSB + 7 % NaCl	*T. devorans* (100) - JQ799100.1		-	-	-	-	-	-	-	-	-	-	-
** VM 80**	TSB + 3.5 % NaCl	*B. infantis* (99) - KM077280.1	18	13 ± 1.2	13 ± 1.2	13 ± 1.2	38 ± 3.5	29 ± 8.3	19 ± 8.1	+	-	-	-	-
** VM 81**	TSB + 3.5 % NaCl	*B. infantis* (99) - KJ911913.1	4	54 ± 7.2	47 ± 4.6	42 ± 3.5	27 ± 5.8	25 ± 4.6	19 ± 2.3	-	-	-	-	-
**Dois Rios Beach**														
DR 1.0	TSB	*B. niacini* (99) - KJ542777.1		-	-	-	-	-	-	-	-	-	-	-
** DR 2.0**	TSB	*B. asahii* (99) - JN084129.1	7	-	-	-	32 ± 2.8	19 ± 9	14 ± 3.5	-	-	-	-	-
DR 3.0	MB	*Fictibacillus barbaricus* (99) - KJ575018.1		-	-	-	-	-	-	-	-	-	-	-
** DR 4.0**	MB	*F. barbaricus* (99) - KC778367.1	6	63 ± 2.3	63 ± 2.3	61 ± 1.2	-	-	-	-	-	+	-	-
**Massambaba Beach**														
** MA 1.0**	MB	*B. subtilis* (99) - KJ668821.1	1	-	-	-	56 ± 6.4	46 ± 7.8	43 ± 4.2	+	2.3	+	+	-
** MA 2.0**	MB	*B. aquimaris* (99) - EU624438.1	8	-	-	-	56 ± 2.1	31 ± 5.2	30 ± 3.5	-	-	-	+	-
MA 2.1	MB	*B. aquimaris* (99) - KM677908.1		-	-	-	-	-	-	-	-	+	+	-
** MA 3.0**	MB	*B. subtilis* (100) - KP100527.1	1	-	-	-	54 ± 3	46 ± 4.6	46 ± 4.6	+	2	+	+	+
** MA 3.1**	MB	*B. subtilis* (100) - KP100527.1	3	28 ± 4.9	25 ± 1.7	24 ± 1.2	45 ± 0	44 ± 1.7	40 ± 5.2	-	1.5	+	+	+
** MA 5.0**	MB	*B. subtilis* (100) - KP100527.1	2	34 ± 6	34 ± 6	32 ± 3.5	-	-	-	+	2.5	+	+	-
** MA 5.1**	MB	*B. tequilensis* (99) - KC851826.1	1	48 ± 5.5	26 ± 2.9	26 ± 2.9	27 ± 1.7	17 ± 6	17 ± 6	+	1.5	+	+	-
MA 6.0	MB	*B. aquimaris* (100) - KM677908.1		-	-	-	-	-	-	-	-	-	-	-
MA 6.1	MB	*B. aquimaris* (99) - KM677908.1		14 ± 0	13 ± 1.7	10 ± 3.5	-	-	-	-	-	+	+	-
** MA 7.0**	MB	*B. subtilis* (99) - KP100527.1	3	26 ± 2.9	-	-	48 ± 4.2	41 ± 13.8	35 ± 9.2	+	1.7	+	+	-
** MA 7.1**	MB	*B. subtilis* (99) - KJ668821.1	1	36 ± 3.5	30 ± 3.5	30 ± 3.5	13 ± 1.7	-	-	+	3	+	+	-
** MA 8.0**	TSB	*B. subtilis* (99) - KP100527.1	3	30 ± 2.1	27 ± 1.7	25 ± 2.9	18 ± 4.6	14 ± 7.9	11 ± 5.2	+	1.7	+	+	-
MA 9.0	MB	*B. aquimaris* (100) -KM677908.1		-	-	-	-	-	-	-	-	-	+	-
MA 9.1	MB	*B. aquimaris* (100) -KM677908.1		-	-	-	-	-	-	-	-	+	+	-
** MA 10.0**	MB	*B. subtilis* (100) -KP100527.1	1	9 ± 1.7	8 ± 0	-	45 ± 4.6	44 ± 3.5	35 ± 1.7	+	1.9	+	+	-
** MA 10.1**	MB	*B. subtilis* (100) -KP100527.1	1	-	-	-	55 ± 1.7	49 ± 7.1	46 ± 4.6	+	1.8	+	+	-
** MA 11.0**	MB	*B. subtilis* (99) -KP100527.1	3	-	-	-	46 ± 2.9	38 ± 8.7	34 ± 6	+	3.1	+	+	-
** MA 11.1**	MB	*B. subtilis* (99) -KP100527.1	3	-	-	-	44 ± 1.2	44 ± 1.7	30 ± 1.7	+	2	+	-	-
MA 12.0	MB	*B. subtilis* (99) -KF601955.1		-	-	-	14 ± 0	7 ± 4	7 ± 4	+	2.5	+	+	-
** MA 12.1**	MB	*B. subtilis* (100) -KP100527.1	2	-	-	-	54 ± 3	50 ± 3.5	44 ± 1.7	+	1.6	+	+	-
** MA 13.0**	MB	*B. subtilis* (100) KP100527.1	2	-	-	-	51 ± 0	25 ± 5.5	19 ± 6.9	+	2.0	+	+	-
** MA 13.1**	MB	*B. tequilensis* (99) - KC851826.1	1	4 ± 1.2	-	-	53 ± 4.6	48 ± 5.5	44 ± 1.7	+	2.7	+	+	-
** MA 14.0**	MB	*B. subtilis* (100) KP100527.1	3	8 ± 0	8 ± 0	-	46 ± 2.9	32 ± 3.5	28 ± 0	+	2.5	+	+	-
** MA 14.1**	MB	*B. subtilis* (100) KP100527.1	3	-	-	-	53 ± 1.7	45 ± 0	44 ± 1.7	+	1.1	+	+	-
** MA 15.0**	TSB + 3.5 % NaCl	*B. subtilis* (100) KP100527.1	1	36 ± 7.5	31 ± 6	31 ± 6	18 ± 3.5	12 ± 4.6	12 ± 4.6	+	2	+	+	-
** MA 15.1**	TSB + 3.5 % NaCl	*B. licheniformis* (100) - KP052704.1	11	32 ± 3.5	25 ± 2.9	23 ± 0	22 ± 4.6	22 ± 4.6	23 ± 5.2	+	1.6	+	+	+
** MA 16.0**	TSB + 3.5 % NaCl	*B. tequilensis* (99) - KC851826.1	1	20 ± 0	20 ± 0	19 ± 1.7	51 ± 4.2	48 ± 4.2	43 ± 4.2	+	2.2	+	+	-
** MA 16.1**	TSB + 3.5 % NaCl	*B. subtilis* subsp. *subtilis* (99) – KM972669.1	3	12 ± 3.5	12 ± 3.5	11 ± 4.6	46 ± 4.6	26 ± 7.1	18 ± 6.9	+	2.6	+	+	-
**Abraão Beach**														
AB 1.0	TSB + 3.5 % NaCl	*B. subtilis* subsp. *subtilis* (99) – KM972669.1		-	-	-	-	-	-	+	1.8	+	+	-
** AB 1.1**	TSB + 3.5 % NaCl	*B. subtilis* subsp. *subtilis* (99) – KM972669.1	3	14 ± 0	10 ± 3.5	10 ± 3.5	51 ± 0	37 ± 4.2	29 ± 3.5	+	2.5	+	+	-
** AB 2.0**	TSB + 3.5 % NaCl	*B. subtilis* (100) KP100527.1	1	46 ± 2.9	32 ± 1.7	28 ± 0	59 ± 1.7	53 ± 3.5	53 ± 3.5	+	2	+	+	+
AB 3.0	TSB + 3.5 % NaCl	*B. aquimaris* (100) - KM516787.1		-	-	-	-	-	-	-	-	-	-	-
AB 4.0	TSB + 3.5 % NaCl	*B. aquimaris* (99) - KM516787.1		-	-	-	19 ± 1.7	-	-	-	-	-	-	-
**Saltern 1**														
SA1 1	TSB	*F. barbaricus* (100) - KJ831620.1		-	-	-	-	-	-	-	-	+	+	-
SA1 2	TSB + 7 % NaCl	*F. barbaricus* (100) - KJ831620.1		-	-	-	-	-	-	-	-	+	-	-
SA1 3	TSB + 7 % NaCl	*F. barbaricus* (100) - KJ831620.1		-	-	-	-	-	-	-	-	-	-	-
SA1 4	TSB + 7 % NaCl	*F. barbaricus* (98) -KJ575018.1		-	-	-	-	-	-	-	-	-	-	-
SA1 5	MB	*F. barbaricus* (100) - KJ831620.1		-	-	-	-	-	-	-	-	-	-	-
SA1 6	MB	*B. oceanisediminis* (100) - KM873030.1		-	-	-	-	-	-	-	-	+	-	-
SA1 7	MB	*B. firmus* (99) - KP119100.1		-	-	-	-	-	-	-	-	-	-	-
SA1 8	MB	*B. firmus* (99) - KF886286.1		-	-	-	-	-	-	-	-	-	-	-
** SA1 10**	MB	*B. firmus* (99) - KP119100.1	2	32 ± 3.5	-	-	49 ± 1.7	-	-	-	-	+	-	-
SA1 11	MB	*F. barbaricus* (100) - KJ831620.1		-	-	-	-	-	-	-	-	+	-	-
SA1 12	MB	*F. barbaricus* (99) - KJ831620.1		-	-	-	-	-	-	-	-	+	-	-
SA1 13	MB	*F. barbaricus* (100) - KJ831620.1		-	-	-	-	-	-	-	-	+	-	-
** SA1 14**	MB	*B. firmus* (100) -KP119100.1	2	-	-	-	51 ± 7.4	-	-	-	-	+	-	-
** SA1 15**	MB	*B. firmus* (100) - KJ528874.1	2	-	-	-	52 ± 7.1	-	-	-	-	+	-	-
** SA1 16**	MB	*B. firmus* (99) - KF886286.1	2	38 ± 3.5	38 ± 3.5	14 ± 0	52 ± 1.7	13 ± 1.7	-	-	-	-	-	-
** SA1 17**	MB	*B. firmus* (99) - KP119100.1	2	43 ± 2.9	-	-	48 ± 0	17 ± 5.2	-	-	-	-	-	-
** SA1 18**	TSB + 7 % NaCl	*B. firmus* (99) - KP119100.1	2	41 ± 1.7	14 ± 0	-	46 ± 2.9	15 ± 1.7	-	-	-	-	-	-
SA1 19	TSB + 7 % NaCl	*B. niabensis* (99) -KF861607.1		-	-	-	-	-	-	-	-	-	-	-
**Saltern 2**														
** SA2 1**	MB	*B. oceanisediminis* (100) - KM873030.1	5	39 ± 1.7	14 ± 0	-	51 ± 4.2	-	-	-	-	-	-	-
** SA2 2**	MB	*B. firmus* (100) - KP119100.1	5	36 ± 1.7	7 ± 4	7 ± 4	50 ± 6.4	-	-	-	-	-	-	-
** SA2 3**	MB	*B. firmus* (100) - KP119100.1	2	-	-	-	65 ± 2.9	44 ± 10	38 ± 6.9	-	-	-	-	-
** SA2 4**	MB	*B. firmus* (100) - KP119100.1	5	-	-	-	51 ± 7.4	43 ± 4.6	42 ± 3.5	-	-	-	-	-
** SA2 5**	MB	*B. firmus* (100) - KP119100.1	2	-	-	-	51 ± 4.2	-	-	-	-	-	-	-
SA2 6	MB	*B. firmus* (100) - KP119100.1		-	-	-	-	-	-	-	-	-	-	-
** SA2 7**	MB	*B. firmus* (100) - KP119100.1	2	-	-	-	51 ± 8.5	38 ± 7	-	-	-	-	-	-
** SA2 8**	MB	*B. oceanisediminis* (100) - KM873030.1	2	45	-	-	-	-	-	-	-	-	-	-
** SA2 9**	MB	*B. oceanisediminis* (99) - KM873030.1	2	23 ± 8.1	-	-	51 ± 7.4	48 ± 4.6	-	-	-	-	-	-
**Saltern 2 (mud)**														
LS 1	TSB + 7 % NaCl	*B. horikoshii* (100) - KJ722424.1		-	-	-	-	-	-	-	-	-	-	-
LS 2	TSB + 7 % NaCl	*B. horikoshii* (100) - KJ722424.1		-	-	-	-	-	-	-	-	-	-	-
LS 3	TSB + 7 % NaCl	*B. horikoshii* (100) - KJ722424.1		-	-	-	-	-	-	-	-	-	-	-
LS 4	TSB + 7 % NaCl	*B. horikoshii* (100) - KJ722424.1		-	-	-	-	-	-	-	-	+	-	-
LS 5	TSB + 7 % NaCl	*B. horikoshii* (99) - KJ719363.1		-	-	-	-	-	-	-	-	-	-	-
LS 6	MB	*B. vietnamensis* (99) - JF411279.1		-	-	-	-	-	-	-	-	+	-	-
LS 7	MB	*B. vietnamensis* (99) - JF411279.1		-	-	-	-	-	-	-	-	-	-	-
LS 8	MB	*B. vietnamensis* (100) - JF411279.1		-	-	-	-	-	-	-	-	-	-	-
LS 10	MB	*B. vitnamensis* (99) - JF411279.1		-	-	-	-	-	-	-	-	+	-	-
LS 11	MB	*B. foraminis* (97) - KF724906.1		-	-	-	-	-	-	-	-	-	-	-
LS 12	TSB	*B. foraminis* (99) - KF724906.1		-	-	-	-	-	-	-	-	-	-	-
** LS 13**	TSB	*Paenisporosarcina indica* (99) - NR108473.1	13	-	-	-	59 ± 7.1	38 ± 7	-	-	-	-	-	-
LS 14	TSB	*P. quisquiliarum* (99) - JF309238.1		-	-	-	-	-	-	-	-	-	-	-
LS 15	TSB	*P. quisquiliarum* (99) - JF309238.1		-	-	-	-	-	-	-	-	-	-	-
LS 16	TSB	*B. licheniformis* (100) - LN774355.1		-	-	-	-	-	-	-	-	-	-	+
LS 17	TSB + 3.5 % NaCl	*B. aquimaris* (99) - JF411268.1		-	-	-	-	-	-	-	-	-	-	-
LS 18	TSB + 3.5 % NaCl	*P. indica* (99) - NR108473.1		-	-	-	-	-	-	-	-	-	-	-
LS 19	TSB + 3.5 % NaCl	*B. thioparans* (99) - KJ911915.1		-	-	-	-	-	-	-	-	-	-	-
LS 20	TSB + 3.5 % NaCl	*B. foraminis* (99) -KF724906.1		-	-	-	-	-	-	-	-	-	-	-
LS 21	TSB + 3.5 % NaCl	*B. foraminis* (99) -KF724906.1		-	-	-	-	-	-	-	-	-	-	-

^a^ First hit in GenBank with a known species; *EI* emulsification index, *Hex* hexadecane, *Ker* kerosene, *DO* drop collapse, *OD* oil displacement, *H* hemolysis, *sfp* presence of *sfp* gene, *AMS* production of an antimicrobial substance. (−) negative results. Strains in bold are those showing emulsification values higher than 30 %

### Biosurfactant production

Emulsification assays were performed using either hexadecane or kerosene. More than half of the strains isolated were able to emulsify at least one of the hydrocarbons used. In contrast, 95 % and 66.7 % of the isolates did not show emulsification activity in particular environments such as Saline 2 mud and Saline 1 (hypersaline samples), respectively (Table 2). In total, 52 of the 110 isolated strains presented an EI % higher than 30 % and were chosen for further studies (11 from Vermelha Lagoon, 2 from Dois Rios Beach, 22 from Massambaba Beach, 2 from Abraão Beach, 6 from Saltern 1, 8 from Saltern 2 and only one from saltern 2 mud; Table 2). The great majority of the strains presenting EI % higher than 30 % belonged to the genus *Bacillus. B. infantis* was predominant in Vermelha Lagoon, *B. subtilis* in Massambaba Beach and *B. firmus* in both salterns. Strains belonging to other genera were only found in Dois Rios Beach (*F. barbaricus* – one strain) and in Saltern 2 mud (*P. indica* – one strain).

### Drop-collapse and oil spreading tests, hemolytic activity and production of antimicrobial substances (AMS)

All strains that did not show emulsification activity also gave negative results in the drop-collapse and oil spreading tests. Only 26.4 % and 22.7 % of the 110 strains were positive in the drop-collapse and oil spreading tests, respectively (Table 2). Strains (39.1 %) belonging to different species presented hemolytic activity in 5 % sheep blood agar plates. *Bacillus subtilis*, *B. licheniformis*, *B. infantis*, *B. aquimaris*, *B. tequilensis* and *F. barbaricus* were species that predominantly presented hemolytic activity (Table 2).

Different biosurfactants produced by the aerobic endospore-forming bacteria usually present two properties (surfactant and antimicrobial); thus, all isolates were screened for the production of antimicrobial compounds against *Micrococcus* sp. Only 10 % of the strains (mainly isolated from Massambaba Beach, Vermelha Lagoon and Saltern 2 mud) inhibited the growth of the indicative strain (Table 2).

### Detection of *sfp* gene coding for surfactin production

The presence of the gene *sfp* coding for surfactin production was also screened among the isolates. The gene *sfp* was found in 32.7 % of the isolates (Table 2), including endospore-forming bacteria related to different species from the genus *Bacillus* and to *Halobacillus trueperi* and *H. mangrove* (Table 2). DNA sequencing analysis showed that the sequences of the gene *sfp* obtained from all *Bacillus* strains shared 99 % identity with the *sfp* gene of *B. subtilis*, whereas the *sfp* sequences of *Halobacillus trueperi* and *H. mangrove* shared only 86 % and 97 % sequence identity, respectively, with those of the *B. subtilis sfp* gene (data not shown).

### Emulsion values (%) of the supernatant of one representative strain of each BOX group at high temperatures and in different amounts of NaCl

**Fig. 5:**
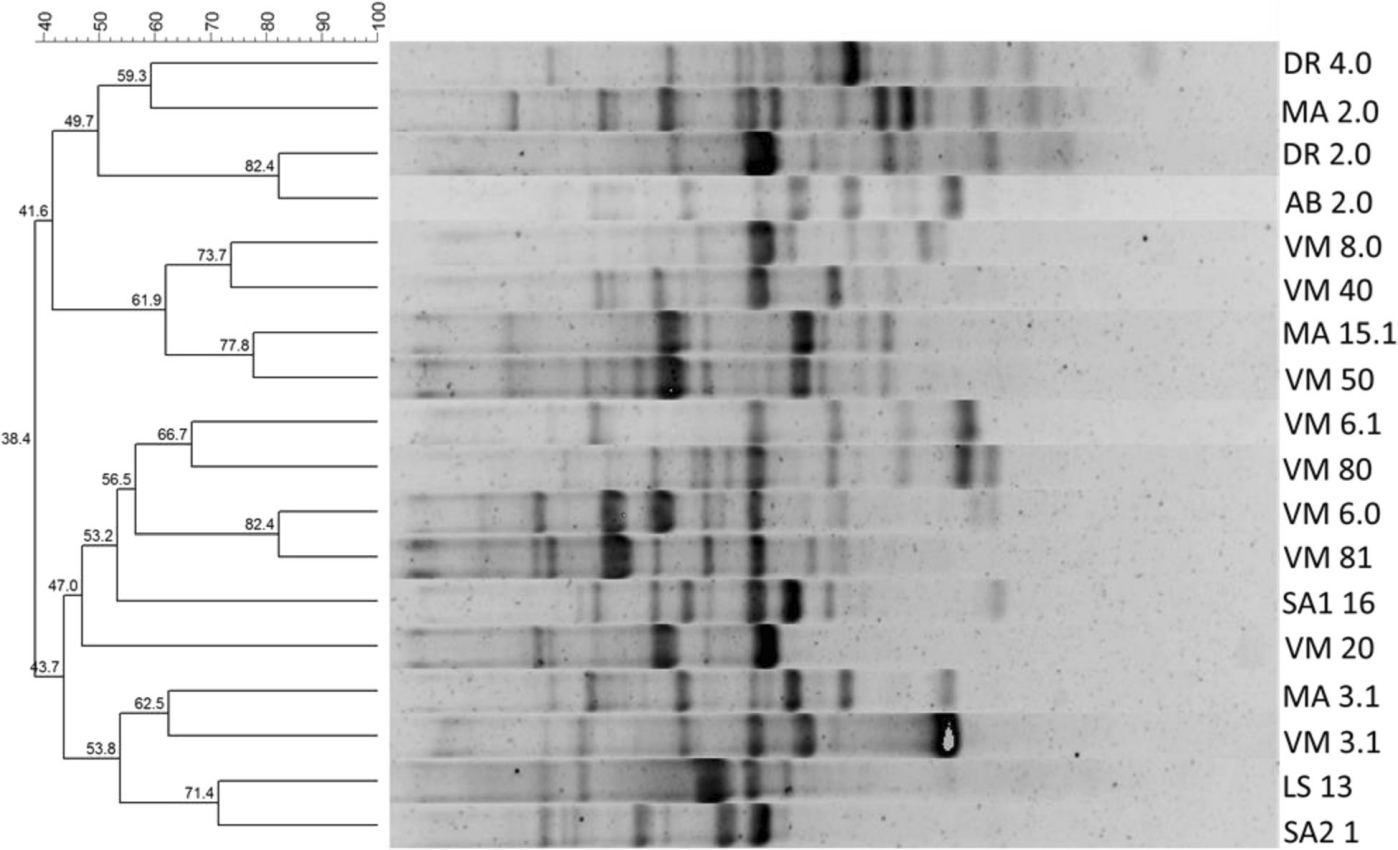
Dendrogram showing representative strains of each 18 groups formed via BOX-PCR

The 52 strains, which presented an EI % higher than 30 %, were divided into 18 groups (90 % similarity) according to the BOX dendrogram (Fig. 5, Table 2). Some groups were formed by more than 9 strains (groups 1, 2 and 3) and others by only one strain (groups 6 to 18; Table 2). One representative strain from each group was selected for testing the stability of their biosurfactants after the treatment with salt and heat.

Emulsion values (%) of representative strains using kerosene were usually higher than those obtained with hexadecane at 100 °C. The stability of the biosurfactants at 121 °C was usually very low. Only two strains still showed an EI % higher than zero - VM 40 and MA 15.1 - using hexadecane (Table 3). However, the biosurfactant produced by strain VM 40 (*B. infantis*) and tested with kerosene was stable for 30 days after being treated at 121 °C (Table 3).

**Table 3:** Emulsion indices (%) of one representative strain of each BOX group using kerosene or hexadecane and high temperature

		Emulsion index (%)			Emulsion index (%)			Emulsion index (%)			Emulsion index (%)		
		Hexadecane - 100 °C			Hexadecane - 121 °C			Kerosene - 100 °C			Kerosene - 121 °C		
Strains	BOX group	1 day	15 days	30 days	1 day	15 days	30 days	1 day	15 days	30 days	1 day	15 days	30 days
AB 2.0	1	46 ± 2.9	43 ± 0	40 ± 0	-	-	-	52 ± 8.6	51 ± 0	36 ± 7.5	-	-	-
SA1 16	2	-	-	-	-	-	-	-	-	-	-	-	-
MA 3.1	3	52 ± 7.8	49 ± 7.8	49 ± 7.8	-	-	-	48 ± 10.3	49 ± 7.8	32 ± 8.6	6 ± 1.7	-	-
VM 81	4	-	-	-	-	-	-	39 ± 1.7	-	-	-	-	-
SA2 1	5	-	-	-	-	-	-	-	-	-	-	-	-
DR 4.0	6	-	-	-	-	-	-	-	-	-	-	-	-
DR 2.0	7	-	-	-	-	-	-	56 ± 1.7	53 ± 4.6	40 ± 7.9	50 ± 1.7	-	-
MA 2.0	8	-	-	-	-	-	-	47 ± 9.9	30 ± 6.4	-	26 ± 2.9	-	-
VM 8.0	9	63 ± 5.2	-	-	-	-	-	50 ± 2.1	-	-	-	-	-
VM 40	10	50 ± 1.7	36 ± 10.6	29 ± 7.8	13 ± 1.7	-	-	55 ± 3.5	52 ± 8.1	48 ± 0	53 ± 2.1	48 ± 0	42 ± 2.1
MA 15.1	11	39 ± 9.6	39 ± 9.6	-	8 ± 0	8 ± 0	8 ± 0	44 ± 3.5	32 ± 3.5	10 ± 7	-	-	-
VM 50	12	-	-	-	-	-	-	49 ± 5.7	30 ± 9.9	-	-	-	-
LS 13	13	-	-	-	-	-	-	-	-	-	-	-	-
VM 3.1	14	-	-	-	-	-	-	-	-	-	-	-	-
VM 6.0	15	40 ± 5.2	-	-	-	-	-	37 ± 9.0	37 ± 12.7	-	36 ± 6.9	-	-
VM 6.1	16	-	-	-	-	-	-	-	-	-	-	-	-
VM 20	17	58 ± 4.6	42 ± 10.1	26 ± 8.6	-	-	-	59 ± 1.7	56 ± 2.1	47 ± 5.7	-	-	-
VM 80	18	-	-	-	-	-	-	-	-	-		-	-

When different concentrations of salt were added to the supernatant of the biosurfactant producing strains, only two strains (AB 2.0 and MA 3.1) showed emulsification with up to 10 % NaCl using hexadecane (Table 4). Considering the same test but using kerosene as the hydrocarbon chosen, strains DR 2.0, VM 8.0, VM 40, VM 6.0 and VM 20 showed promising results with up to 20 % NaCl added to the supernatants (Table 5). Particularly, strain DR 2.0 (*B. asahii*) was stable throughout the experiment (30 days) after the addition of this high amount of salt (Table 5).

**Table 4:** Emulsion indices (%) of one representative strain of each BOX group using hexadecane and different amounts of NaCl

	Emulsion index (%)			Emulsion index (%)			Emulsion index (%)			Emulsion index (%)		
	Hexadecane – NaCl 5 %			Hexadecane – NaCl 10 %			Hexadecane – NaCl 15 %			Hexadecane – NaCl 20 %		
Strains	1 day	15 days	30 days	1 day	15 days	30 days	1 day	15 days	30 days	1 day	15 days	30 days
AB 2.0	46 ± 10.8	24 ± 1.7	9.5 ± 2.1	45 ± 7.1	14 ± 0	10 ± 2.1	-	-	-	-	-	-
SA1 16	-	-	-	-	-	-	-	-	-	-	-	-
MA 3.1	52 ± 5.7	12 ± 3.5	7.3 ± 4	50 ± 10	26 ± 2.9	26 ± 2.9	19 ± 7.5	-	-	-	-	-
VM 81	-	-	-	-	-	-	-	-	-	-	-	-
SA2 1	-	-	-	-	-	-	-	-	-	-	-	-
DR 4.0	-	-	-	-	-	-	-	-	-	-	-	-
DR 2.0	-	-	-	-	-	-	-	-	-	-	-	-
MA 2.0	-	-	-	-	-	-	-	-	-	-	-	-
VM 8.0	-	-	-	-	-	-	-	-	-	-	-	-
VM 40	56 ± 7.5	-	-	-	-	-	-	-	-	-	-	-
MA 15.1	-	-	-	-	-	-	-	-	-	-	-	-
VM 50	-	-	-	-	-	-	-	-	-	-	-	-
LS 13	-	-	-	-	-	-	-	-	-	-	-	-
VM 3.1	-	-	-	-	-	-	-	-	-	-	-	-
VM 6.0	-	-	-	-	-	-	-	-	-	-	-	-
VM 6.1	-	-	-	-	-	-	-	-	-	-	-	-
VM 20	23 ± 0	17 ± 8.5	-	-	-	-	-	-	-	-	-	-
VM 80	-	-	-	-	-	-	-	-	-	-	-	-

**Table 5:** Emulsion indices (%) of one representative strain of each BOX group using kerosene and different amounts of NaCl

	Emulsion index (%)			Emulsion index (%)			Emulsion index (%)			Emulsion index (%)		
	kerosene – NaCl 5 %			kerosene – NaCl 10 %			kerosene – NaCl 15 %			kerosene – NaCl 20 %		
Strains	1 day	15 days	30 days	1 day	15 days	30 days	1 day	15 days	30 days	1 day	15 days	30 days
AB 2.0	53 ± 4.6	22 ± 1.7	10 ± 2.1	49 ± 1.7	12 ± 3.5	10 ± 6.4	-	-	-	-	-	-
SA1 16	-	-	-	-	-	-	-	-	-	-	-	-
MA 3.1	56 ± 6.4	26 ± 3.5	-	-	-	-	-	-	-	-	-	-
VM 81	-	-	-	-	-	-	-	-	-	-	-	-
SA 2 1	-	-	-	-	-	-	-	-	-	-	-	-
DR 4.0	-	-	-	-	-	-	-	-	-	-	-	-
DR 2.0	51 ± 7.6	44 ± 6.4	36 ± 6.2	53 ± 6.4	49 ± 8.1	47 ± 6.4	57 ± 0	54 ± 0	47 ± 1.4	52 ± 3.5	48 ± 7.4	44 ± 8.7
MA 2.0	-	-	-	-	-	-	-	-	-	-	-	-
VM 8.0	51 ± 8.5	41 ± 3.5	33 ± 3.5	51 ± 3	46 ± 0	32 ± 8.1	57 ± 0	48 ± 0	34 ± 0	39 ± 9.6	-	-
VM 40	55 ± 3.5	40 ± 5.2	-	51 ± 4.2	39 ± 2.1	-	59 ± 2.9	51 ± 5.5	-	63 ± 2.5	48 ± 8.1	-
MA 15.1	-	-	-	-	-	-	-	-	-	-	-	-
VM 50	-	-	-	-	-	-	-	-	-	-	-	-
LS 13	-	-	-	-	-	-	-	-	-	-	-	-
VM 3.1	-	-	-	-	-	-	-	-	-	-	-	-
VM 6.0	45 ± 7.4	41 ± 3.5	-	48 ± 0	43 ± 0	-	47 ± 4	45 ± 1.7	-	48 ± 2.5	43 ± 0	-
VM 6.1	-	-	-	-	-	-	-	-	-	-	-	-
VM 20	52 ± 4.6	37 ± 7.9	-	44 ± 9.1	40 ± 10.4	-	43 ± 8.1	37 ± 12.5	-	-	-	-
VM 80	-	-	-	-	-	-	-	-	-	-	-	-

### Detection of surfactin using ESI–MS/MS triple quadrupole mass spectrometry

**Fig. 6:**
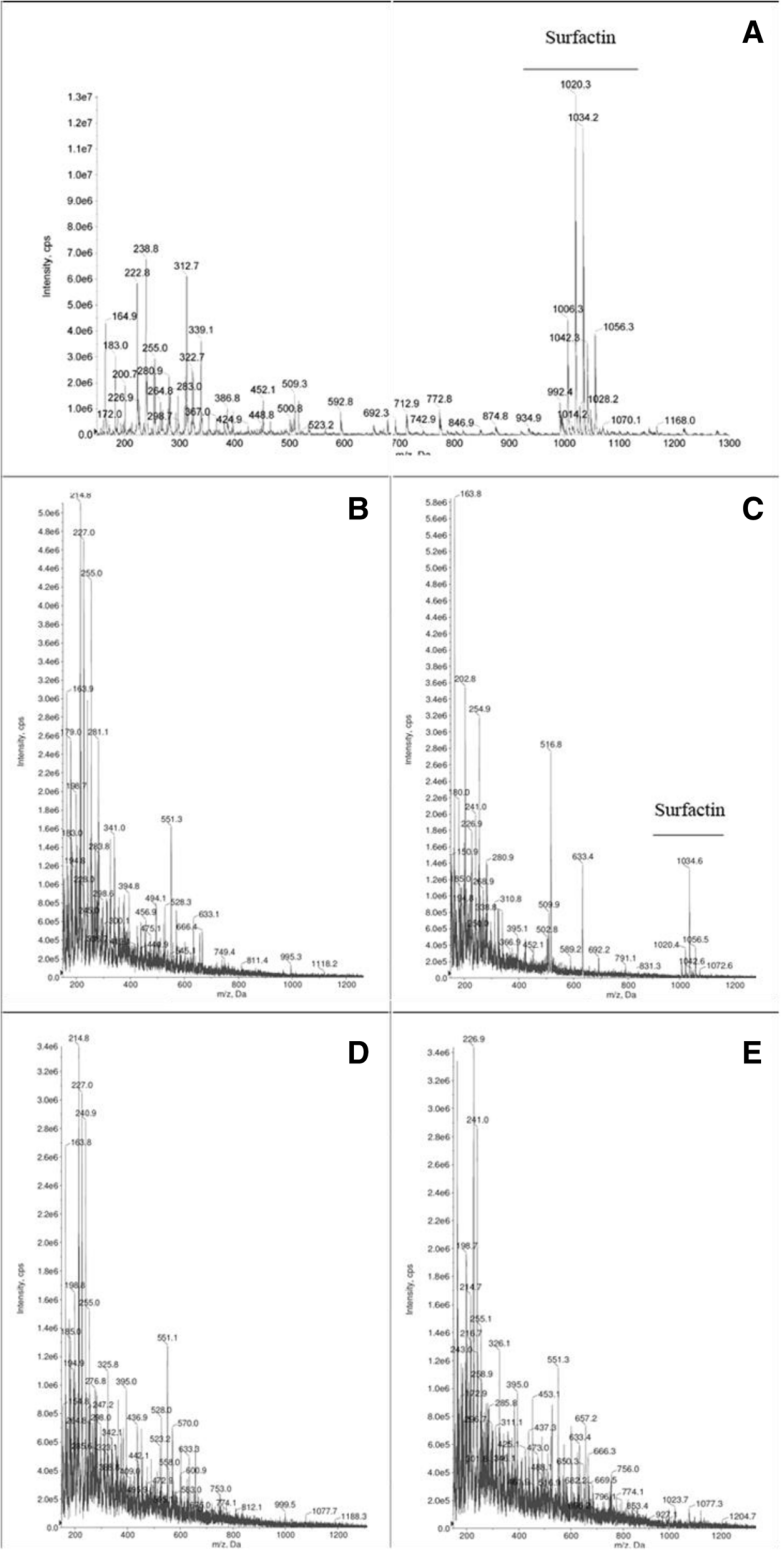
ESI–MS/MS triple quadrupole mass spectrometry in negative ion mode of the crude extracts of four strains: (**a**) Commercial surfactin (Sigma Aldrich) used as positive control, (**b**) B. asahii DR 2.0, (**c**) B. subtilis AB 2.0, (**d**) B. infantis VM 6.0 and (**e**) B. infantis VM 8.0

The production of surfactin was analyzed in four biosurfactant-producing bacteria (DR 2.0, AB 2.0, VM 6.0 and VM 8.0). A cluster of peaks with mass/charge (m/z) ratios between 1020 and 1034, which could be attributed to surfactin isoforms, was observed only in the control (commercial surfactin; Fig. 6a) and in AB 2.0, identified as *B. subtilis* (Fig. 6c).

## Discussion

The importance of endospore-forming bacteria as biosurfactant producers and their presence in saline and hypersaline environments were combined in this study. Both independent (utilizing primers that target bacilli) and dependent cultivation-based approaches were used to achieve our goals. The results obtained here (PCR-DGGE followed by NMDS analyses) showed a diverse endospore-forming bacterial community in all environments and the grouping of the environmental samples based on their salinity. Likewise, Wang et al. [33] demonstrated that salinity was the environmental variable with the strongest influence on the bacterial community composition of 32 pristine Tibetan lakes that represent a broad salinity range (freshwater to hypersaline). The diversity of the endospore-forming bacterial community observed among the environmental samples analyzed supported the choice for a better characterization of the biosurfactant-producing and endospore-forming bacteria in these marine and hypersaline ecosystems.

For cultivation-based methods, we heated all samples to 80 °C for 10 min to facilitate the study of endospore-forming bacterial communities. Therefore, only the spores were addressed, possibly leading to an underestimation of the number of bacilli in the various samples studied here. The bacterial colony counts ranged from 10^3^ to less than 10^5^ CFU g^−1^ of sediment/mud sample, but no direct correlation between the number of colonies and salinity was observed.

From the 110 strains isolated, 52 were considered potential biosurfactant-producing strains, showing good emulsification properties (EI higher than 30 %; Table 2). Good emulsification is critical for biosurfactants to be promising in various environmental and industrial applications [3]. Moreover, other biosurfactants properties, such as drop-collapse and oil spreading, hemolytic and antimicrobial activities were also tested in all isolates. As being previously observed by other authors, we could not observe a close link in these tests. For example, da Silva et al. [34] isolated strains with hemolytic activity; however, only 33 % were confirmed as biosurfactant producers via the oil-spreading method.

Considering the surfactin group as one of the most powerful biosurfactants described so far, we investigated the presence of the gene *sfp* (coding for surfactin in *B. subtilis* [30, 35]) in all isolates. Although many strains showed the expected PCR product (Table 2), the gene does not appear to be expressed in many of the strains: no biosurfactant activities were observed. Furthermore, the gene was detected not only in *Bacillus* strains but also in strains identified as belonging to *Halobacillus* species*.* These *sfp*-related genes remain poorly characterized in genera other than *Bacillus*.

The sequencing of the partial 16SrRNA gene revealed that the isolates belong not only to different species of the genus *Bacillus* but also to *Thalassobacillus*, *Halobacillus*, *Paenibacillus*, *Fictibacillus* and *Paenisporosarcina*. Some *Bacillus* species found in this study are not well studied, and many of them have not been related to biosurfactant production, such as *B. asahii*, *B. detrensis*, and others. Furthermore, to our knowledge, this is the first time *F. barbaricus* and *P. indica* have been described as biosurfactant producers. Strains phylogenetically related to these species (DR 4.0 and LS13) were isolated in Dois Rios Beach and Saltern 2 mud; these presented the lowest and the highest amounts of salt, respectively. The biosurfactant molecules produced by these isolates are possibly new or at least present distinct activities under high salt conditions compared with the others that are already well characterized.

Biosurfactants are usually more effective at a wide range of salinity and temperature values compared with synthetic surfactants [36, 37]. Moreover, both temperature and NaCl are key parameters that affect emulsifying activity and, in microbial-enhanced oil recovery processes, biosurfactants are used as coadjutants with stable agents that are also required due the high temperature, pressure and salinity of the oil reservoirs [38]. Eighteen selected strains (belonging to the different BOX groups) were tested for the loss of emulsifying activity treating the supernatant (cell-free broth) at 100 °C and 121 °C and with different amounts of salt. Reductions in emulsification activity at high temperatures and 10 % NaCl were observed in nearly 80 % (100 °C) and 90 % (121 °C and 10 % NaCl) of the strains using n-hexadecane (measurements made after 30 days). No strains showed emulsification when 15 % NaCl was added to the supernatants. The loss of emulsifier activity could be explained by the denaturation of proteinaceous compounds of the bioemulsifiers during heating. Moreover, once the salinity decreases the viscosity, the increase in NaCl concentration may have influenced the quality of the emulsion, thereby reducing the emulsification capacity. The same loss of emulsifying activity was reported for other microorganism surfactants [39, 40].

Conversely, different strains showed emulsion-stabilization in both temperatures and in up to 20 % NaCl using kerosene. Strains DR 2.0 (*B. asahii*), VM 8.0, VM 40 and VM 6.0 (all *B. infantis*) maintained their emulsification activities for at least 15 days (with the exception of VM 8.0, which lasted only 24 h) with the addition of 20 % NaCl to their supernatants, thereby showing a high tolerance to the salt concentrations. No surfactins were detected in these strains using ESI–MS/MS triple quadrupole mass spectrometry; thus, this study provides a new perspective to better characterize these isolates and their biosurfactants.

## Conclusion

Molecular approaches showed the structural diversity of endospore-forming bacterial communities in different Brazilian marine and hypersaline environments from an Environmental Protection Area located in Rio de Janeiro based on the salinity of the environments. Therefore, these environmental samples were considered to be particularly interesting for the isolation of potential biosurfactant producers belonging to this bacterial group. Different endospore-forming bacterial genera were isolated, but *Bacillus* spp. predominated in all the saline and hypersaline samples. For the first time, *B. asahii*, *B. detrensis*, *F. barbaricus* and *P. indica* were described as biosurfactant producers. The use of biosurfactants produced by endospore-forming bacterial isolates showing emulsification properties in high temperatures and/or in up to 20 % NaCl could be extremely valuable not only for a possible bioremediation approach in saline and hypersaline environments but also for microbial-enhanced oil recovery (MEOR).
